# Exploration of the medial border for laparoscopic D3 lymph node dissection in right hemi-colon cancer: a systematic review and meta-analysis

**DOI:** 10.3389/fsurg.2025.1651549

**Published:** 2025-10-27

**Authors:** Minchen Wang, Zhiyuan Li, Xin Fan, Jixiang Chen, He Han

**Affiliations:** Department of Gastrointestinal Surgery, Affiliated Hospital of Jiangsu University, Zhenjiang, China

**Keywords:** medial border, laparoscopic, D3 lymph node dissection, right hemi-colon cancer, meta-analysis

## Abstract

**Introduction:**

The optimal medial boundary for lymph node dissection during laparoscopic radical right hemicolectomy for colorectal cancer remains uncertain. We investigated whether the superior mesenteric artery (SMA) or superior mesenteric vein (SMV) should serve as the medial border for D3 lymph node dissection in right hemicolon cancer.

**Methods:**

We systematically searched the Cochrane Library, EMBASE, CNKI, VIP, Wanfang, ClinicalTrials.gov, and SinoMed databases through March 2024. Studies comparing SMA- and SMV-based medial borders were included according to predefined criteria. Outcomes analyzed included intraoperative parameters, postoperative recovery, lymph node yield, complications, and survival.

**Results:**

Compared with the SMV group, the SMA group had more lymph nodes cleared (*P* = 0.001) and more positive nodes retrieved (*P* = 0.04), but also longer postoperative drain placement (*P* = 0.01). Intraoperative bleeding was higher in the SMV group (*P* = 0.01). Meta-analysis of patients’ postoperative overall survival (*P* = 0.927) and recurrence-free survival (*P* = 0.949) showed no significant differences in short-term prognosis between the two groups.

**Discussion:**

Using the SMA's left border for laparoscopic D3 dissection is safe and feasible, providing higher lymph node yield without increasing major complications. However, this greater yield did not translate into improved short-term survival. The long-term prognostic effect of the SMA approach requires further investigation.

**Systematic Review Registration:**

identifier (CRD42024502882).

## 1 Introduction

According to Colorectal Cancer Statistics 2023, colorectal cancer (CRC) is the third leading cause of morbidity and mortality in the United States (US) (1); it ranks second in China based on data from the China Cancer Center (2). Laparoscopic radical right hemicolectomy has been a primary surgical method for right-sided colon cancer since the introduction of D3 lymph node dissection in Japan (1976). The concept of complete mesocolic excision (CME) was subsequently proposed by Hohenberger in 2009 (3). he 2020 Japanese Convention on the Management of Colorectal Cancer outlines D3 lymph node dissection principles (4). However, the medial boundary of lymph node dissection for laparoscopic radical right hemicolectomy—whether based on CME or D3 principles—remains unclear.

For safety, the left side of the superior mesenteric vein (SMV) is widely accepted as the medial boundary for D3 lymph node dissection in right-sided colon cancer (5), with approximately 75% of surgeons surveyed endorsing this approach (5). Nevertheless, lymph nodes are also distributed around the superior mesenteric artery (SMA) (6), and the central lymph node group resides near the origin of this artery. Using the SMV as the boundary may therefore fail to adequately clear lymph nodes at the colic artery origins, potentially compromising central lymph node dissection (4). Consequently, while the SMV boundary remains common, some scholars have proposed using the SMA as the medial boundary for D3 dissection in right hemicolon cancer, though consensus is lacking.

This study aimed to determine the optimal medial boundary for laparoscopic D3 lymph node dissection in right hemicolon cancer through meta-analysis. The findings could provide higher-level evidence to guide clinical practice.

## 2 Materials and methods

The study followed the Preferred Reporting Items for Systematic Reviews and Meta-Analyses (PRISMA) guidelines (7) and was registered with PROSPERO (CRD42024502882). Ethics board approval and written informed consent were not required because the study data were downloaded from an open database.

### 2.1 Search strategy

Two authors independently conducted literature searches up to March 2024 in databases, including PubMed, Cochrane Library, EMBASE, CNKI, VIP, Wanfang, ClinicalTrials.gov, and SinoMed, without language restrictions. The search strategy followed the Cochrane Handbook, combining subject and free-text terms. The search keywords included “right colon + lymph node” or “right colon + mesentery.” The retrieved studies were screened based on the inclusion and exclusion criteria. We used the PubMed Inspection Strategy: (((((((((Right hemicolon) OR (right colon)) OR (Hepatic Flexure)) OR (Colon, Ascending)) OR (Flexure, Hepatic)) OR (Ascending Colon)) OR (Right Colic Flexure)) OR (Colic Flexure, Right)) OR (right-sided colon)) AND (((mesentery) OR (Mesenteries)) OR (((((((((((((Lymph node dissection) OR (Lymph Node Excision)) OR (Excision, Lymph Node)) OR (Excisions, Lymph Node)) OR (Lymph Node Excisions)) OR (Lymphadenectomy)) OR (Lymphadenectomies)) OR (Lymph Node Dissection)) OR (Dissection, Lymph Node)) OR (Dissections, Lymph Node)) OR (Lymph Node Dissections)) OR (Node Dissection, Lymph)) OR (Node Dissections, Lymph))).

### 2.2 Inclusion and exclusion criteria

Inclusion criteria included (i) controlled clinical studies that have been published at domestic and international, (ii) the study population was patients undergoing surgery for right hemi-colon cancer, (iii) the study compared the medial boundary limits of D3 lymph node dissection in laparoscopic radical right hemicolectomy for cancer, using the SMA and the SMV as the respective reference boundaries, and (iv) at least one of the following indexes was counted in the literature: general information of the patients, the status of the total postoperative complications, operation time, intraoperative bleeding, number of lymph node cleared, number of positive lymph node cleared, intraoperative vascular-related complications, return of bowel function, resumption of water intake time, drainage tube removal time, and total drainage volume of postoperative drainage tube, days of hospitalization, and postoperative survival data. Exclusion criteria included (i) the study participants were not patients with cancer or patients in emergency, (ii) non-controlled studies, (iii) observation indexes were not reported, (iv) complete clinical data were not provided, and there was no response when the first author was contacted, and (v) duplicate literature.

### 2.3 Data extraction and quality assessment

Based on the inclusion and exclusion criteria, two authors read and screened the literature independently, and in cases of disagreement, inclusion was finalized through group discussion. Both authors extracted the data independently and verified the consistency of the extracted data. The extracted data included title, authors, year of publication, year of study, age of patients, sample size, total postoperative complications, operation time, intraoperative bleeding, number of lymph nodes cleared, number of positive lymph nodes cleared, intraoperative vascular-related complications, return of bowel function, resumption of water intake time, drainage tube removal time, total drainage volume of the postoperative drainage tube, days of hospitalization, and postoperative survival data, including overall survival (OS) and recurrence-free survival (RFS). For continuous variables, where median and range were reported in the literature, mean and standard deviation were calculated according to Luo and Wan's method (https://www.math.hkbu.edu.hk/∼tongt/papers/median2mean.html) (8, 9). If the HR and 95% confidence interval (95% CI) of the observations for survival data were not provided directly in the literature, data extraction was performed using Engauge Digitizer 4.1. For the included literature (10, 11), the Cochrane Collaboration's tool for assessing the risk of bias in randomized trials, RoB2, was used to assess the risk of bias.

### 2.4 Statistical analysis

We extracted observations from each study for a meta-analysis to assess the feasibility of using the left side of the SMA as the medial border in laparoscopic D3 lymph node dissection for right hemicolon cancer. Odds ratios (OR) and 95% CIs were calculated for survival analysis. Statistical heterogeneity was examined in the study using the Q-test, and the analysis was performed using a random-effects model (DerSimonian-Laird method) when *I*^2^ ≥ 50% and *P* ≤ 0.05, and a fixed-effects model (inverse-variance method) when *I*^2^ < 50%. Publication bias was assessed using a funnel plot, and a sensitivity analysis was performed to assess the stability of the results. All statistical analyses of data were performed using STATA 18.0, and *P* ≤ 0.05 was considered statistically significant. Given the anticipated clinical heterogeneity (e.g., variations in surgical technique, tumor staging protocols, or patient demographics), subgroup analyses or meta-regression were planned to explore sources of heterogeneity. However, insufficient primary data reporting in included studies precluded these analyses. Consequently, we relied on sensitivity analyses to assess robustness.

## 3 Results

**Figure 1:**
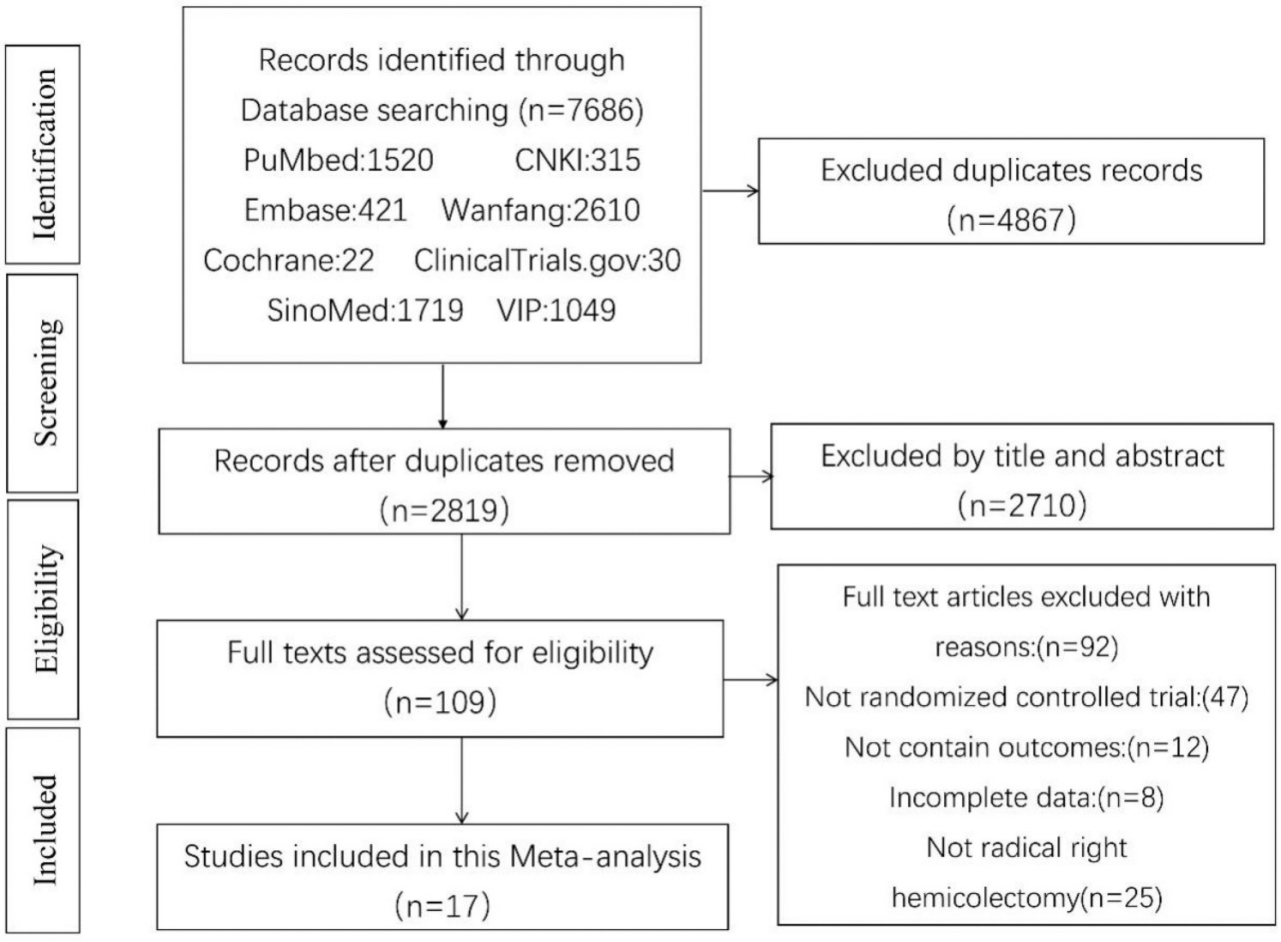
Literature screening process.

According to the search strategy, 7,686 documents with 2,819 duplicates were retrieved. After title and abstract screening, 2,710 publications were excluded, and 109 articles requiring full-text reading were included in the final analysis. During this process, 47 non-randomized, clinically controlled studies were excluded: 12 lacked clinical data from the target study, eight had incomplete data, and 25 had study participants who did not meet the right hemicolon criteria. Ultimately, 17 publications were included in this study (12–28). The screening process is shown in Figure 1.

### 3.1 Characteristics of the included literature

**Figure 2:**
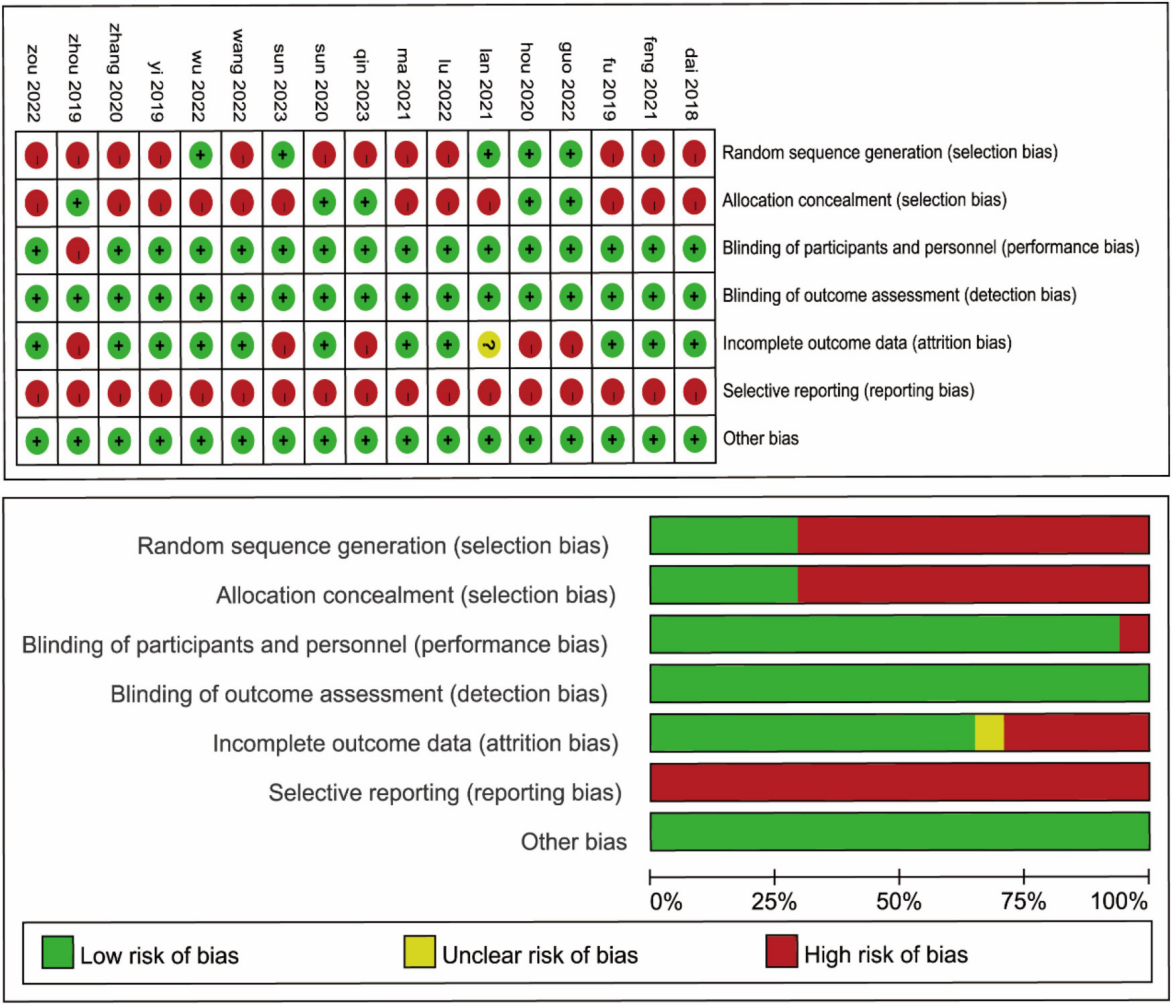
Risk of bias summary of all included studies.

There were 17 publications involving 3,277 patients, of whom 1,351 patients underwent lymph node dissection on the left side of the SMA and 1,926 patients underwent dissection on the left side of the SMV. These studies were RCTs, performed in Asia, and published between 2018 and 2023. Details of the general characteristics of the participants are shown in Table 1. The risk of bias was moderate based on the Cochrane Risk of Bias Assessment (ROB2) tool (Figure 2).

**Table 1:** Characteristics of the included literature.

Study	Year	Research period	Sample size	SMA (Male/Female)	SMV (Male/Female)	Age (years), mean ± SD, SMA/SMV	Study type
Lan et al. (12)	2021	2,016.01–2,019.12	110	55 (30/25)	55 (31/24)	53.61 ± 7.8/22.28 ± 1.8	RCT
Ma et al. (13)	2021	2,017.01–2,018.12	58	30 (20/10)	28 (16/12)	63.5 ± 4.6/62.4 ± 5.2	RCT
Sun et al. (14)	2023	2,018.01–2,020.12	56	28 (12/16)	28 (15/13)	64.3 ± 9.4/63.8 ± 9.6	RCT
Zhou et al. (15)	2019	2,015.06–2,017.03	134	57 (30/27)	77 (42/35)	60.5 ± 10.6/61.3 ± 10.2	RCT
Qin et al. (16)	2023	2,019.09–2,022.08	89	45 (30/15)	44 (24/20)	62.78 ± 5.49/63.15 ± 5.26	RCT
Sun (17)	2020	2,014.12–2,018.03	83	31 (19/12)	52 (27/25)	56.29 ± 12.34/57.12 ± 11.95	RCT
Guo et al. (18)	2022	2,018.12–2,021.12	80	40 (22/18)	40 (20/20)	54.77 ± 3.53/54.68 ± 3.48	RCT
Hou et al. (19)	2020	2,017.05–2,018.12	102	48	54	no	RCT
Wang et al. (20)	2022	2,015.05–2,017.05	108	58 (22/36)	50 (18/32)	59.4 ± 6.3/58.8 ± 6.1	RCT
Sun et al. (21)	2019	2,013.01–2018.06	955	377 (205/172)	578 (309/269)	65.7 ± 13.4/64.3 ± 14.9	RCT
Wu and Yu (22)	2022	2,014.03–2,016.03	80	40 (21/19)	40 (23/17)	58.1 ± 8.1/56.7 ± 9. 8	RCT
Lu and Chen (23)	2022	2,017.02–2,020.01	95	49 (29/20)	46 (27/19)	65.64 ± 8. 51/65.38 ± 8.32	RCT
Sun et al. (24)	2022	2,017.10–2,018.10	60	30 (16/14)	30 (17/13)	67.1 ± 10.2/66.3 ± 10.4	RCT
Zhang et al. (25)	2020	2,015.01–2018.12	76	38 (19/19)	38 (21/17)	51.23 ± 4.19/50.67 ± 4.38	RCT
Zhon et al. (26)	2021	2,013.01–2,018.12	921	307 (169/138)	614 (335/279)	64.6 ± 12.53/64.8 ± 10.2	RCT
Dai et al. (27)	2018	2,010.01–2,014.12	102	34 (16/18)	68 (35/33)	61.9 ± 10.8/61.5 ± 11.2	RCT
Yi et al. (28)	2019	2,017.01–2,018.03	168	84 (40/44)	84 (42/42)	66.05 ± 13.37/64.67 ± 11.86	RCT

SMA, superior mesenteric artery; SMV, superior mesenteric vein; SD, standard deviation; RCT, randomized controlled trial.

### 3.2 Intraoperative-related indicators

**Figure 3:**
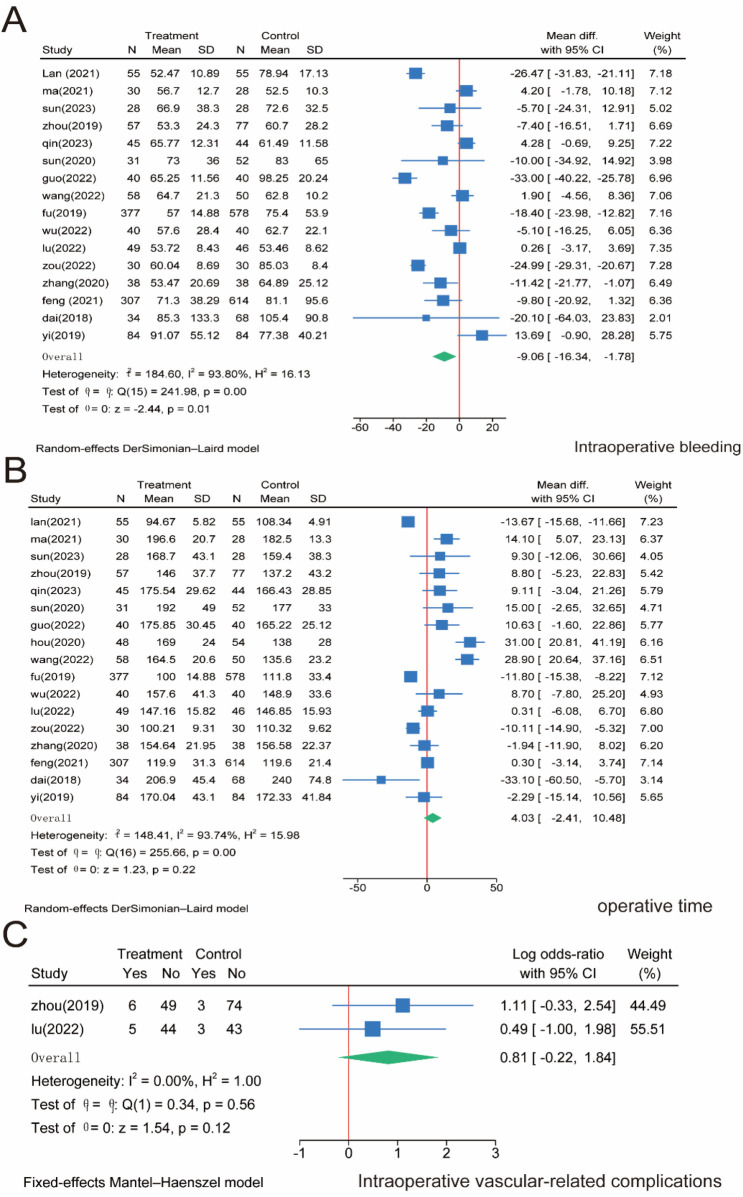
Meta-analysis of intraoperative correlates. **(A)** Intraoperative bleeding. **(B)** Operative time. **(C)** Intraoperative vascular-related complications.

Sixteen papers included 3,175 patients and reported intraoperative bleeding. The meta-analysis showed that intraoperative bleeding was significantly higher in the SMA group than in the SMV group (*P* = 0.01). However, significant heterogeneity was observed between the two groups (*I*^2^ = 93.8%, *P* = 0.01); the significant heterogeneity observed may reflect variations in surgical expertise, patient selection (e.g., BMI, comorbidities), tumor stage distribution, or subtle differences in dissection techniques (e.g., artery-first approach vs. conventional). Sensitivity analysis confirmed result stability, but clinical heterogeneity remains a limitation; therefore, a random effects model was used (Figure 3A). Furthermore, 17 studies with 3,277 patients were included, and the operation time was analyzed in both groups. The results showed no significant difference in operative time between the SMA and SMV groups (*P* = 0.22). Nonetheless, significant heterogeneity was observed between the two groups (*I*^2^ = 93.74%, *P* = 0.00); therefore, a random effects model was used (Figure 3B). Regarding intraoperative vascular complications, only two publications reported relevant indicators and included 229 patients. The results of the meta-analysis showed that there was no significant difference between the two groups of patients (*P* = 0.12), and the heterogeneity between them was insignificant (*I*^2^ = 0.00%, *P* = 0.56); therefore, a fixed-effects model was also used (Figure 3C).

### 3.3 Lymph node-related indicators

**Figure 4:**
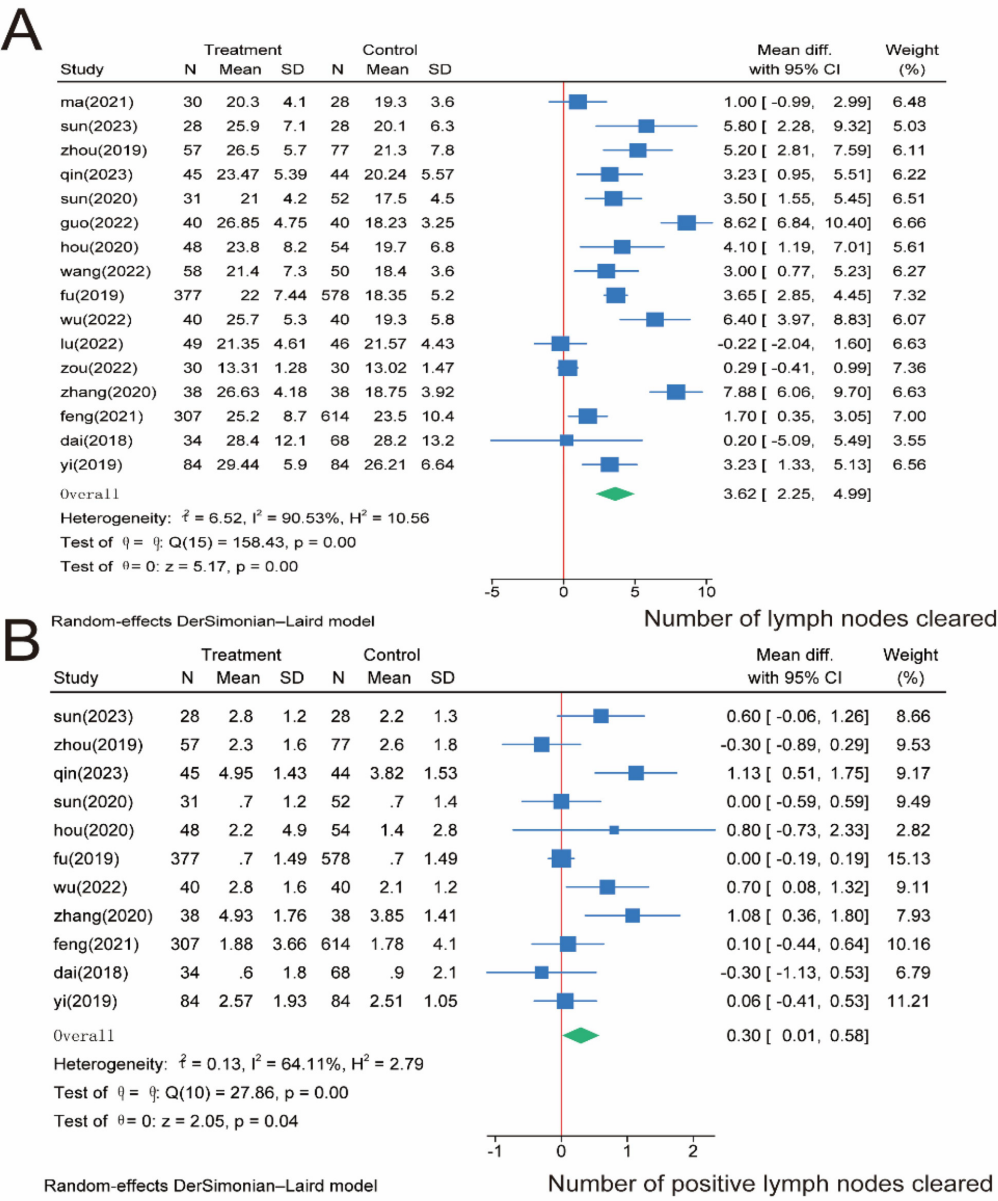
Meta-analysis of lymph node-related indicators. **(A)** Number of lymph nodes cleared intraoperatively. **(B)** Number of positive lymph nodes cleared.

Regarding the number of lymph node dissections, we included 16 studies involving 3,167 patients. The meta-analysis showed that the number of lymph node dissections was significantly higher in the SMA group than in the SMV group (*P* = 0.00). However, significant heterogeneity was observed between the two groups (*I*^2^ = 90.53%, *P* = 0.00); Therefore, we used a random effects model for the meta-analysis of both the total number of lymph nodes cleared (Figure 4A) and the number of positive lymph nodes cleared intraoperatively, which was reported in 11 studies involving 2,766 patients. The meta-analysis showed that the number of positive lymph nodes was significantly higher in the SMA group than in the SMV group (*P* = 0.04). However, significant heterogeneity was observed between the two groups (*I*^2^ = 64.11%, *P* = 0.00); therefore, a random effects model was used (Figure 4B).

### 3.4 Postoperative recovery of patients

**Figure 5:**
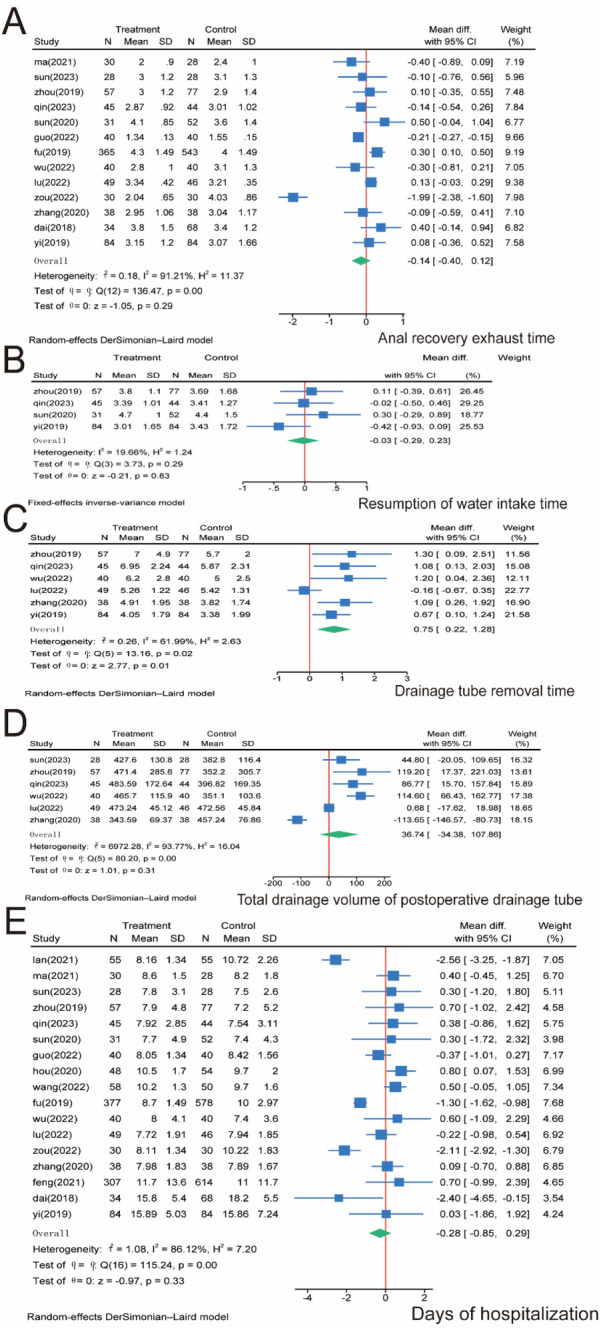
Meta-analysis of patients’ postoperative recovery. **(A)** Return of bowel function. **(B)** Resumption of water intake time. **(C)** Drainage tube removal time. **(D)** Total drainage volume of the postoperative drainage tube. **(E)** Days of hospitalization. N, number; SD, standard deviation; 95% CI, 95% confidence interval.

For patients' return of bowel function, 13 papers involving 1,989 patients were included and analyzed. No significant difference was observed between the SMA and SMV groups regarding time to extubation (*P* = 0.29), and significant heterogeneity was observed between the two groups (*I*^2^ = 91.21%, *P* = 0.00); thus, we used a random effects model (Figure 5A). Regarding the resumption of water intake time, we pooled four studies containing 474 patients. The meta-analysis showed no significant difference between the SMA and SMV groups regarding time to water intake (*P* = 0.83), and the heterogeneity between the two groups was insignificant (*I*^2^ = 19.66%, *P* = 0.29); therefore, a fixed-effects model was used (Figure 5B). Regarding the drainage tube removal time, we included six papers involving 642 patients. The meta-analysis showed significantly longer drainage tube removal time in the SMA group than in the SMV group (*P* = 0.01), and significant heterogeneity was observed between the two groups (*I*^2^ = 61.99%, *P* = 0.02); therefore, a random-effects model was used (Figure 5C). We included six papers involving 530 patients for the total drainage volume of the postoperative drainage tube. The meta-analysis showed no significant difference between the SMA and SMV groups regarding drainage volume (*P* = 0.31), and significant heterogeneity was observed between the two groups (*I*^2^ = 93.77%, *P* = 0.00); therefore, a random-effects model was used (Figure 5D). Finally, after days of hospitalization, we collected 17 papers involving 3,277 patients. The meta-analysis found no significant difference between the SMA and SMV groups regarding days of hospitalization (*P* = 0.33), and significant heterogeneity was observed between the two groups (*I*^2^ = 86.12%, *P* = 0.00); therefore, a random-effects model was used (Figure 5E).

### 3.5 Complications

**Figure 6:**
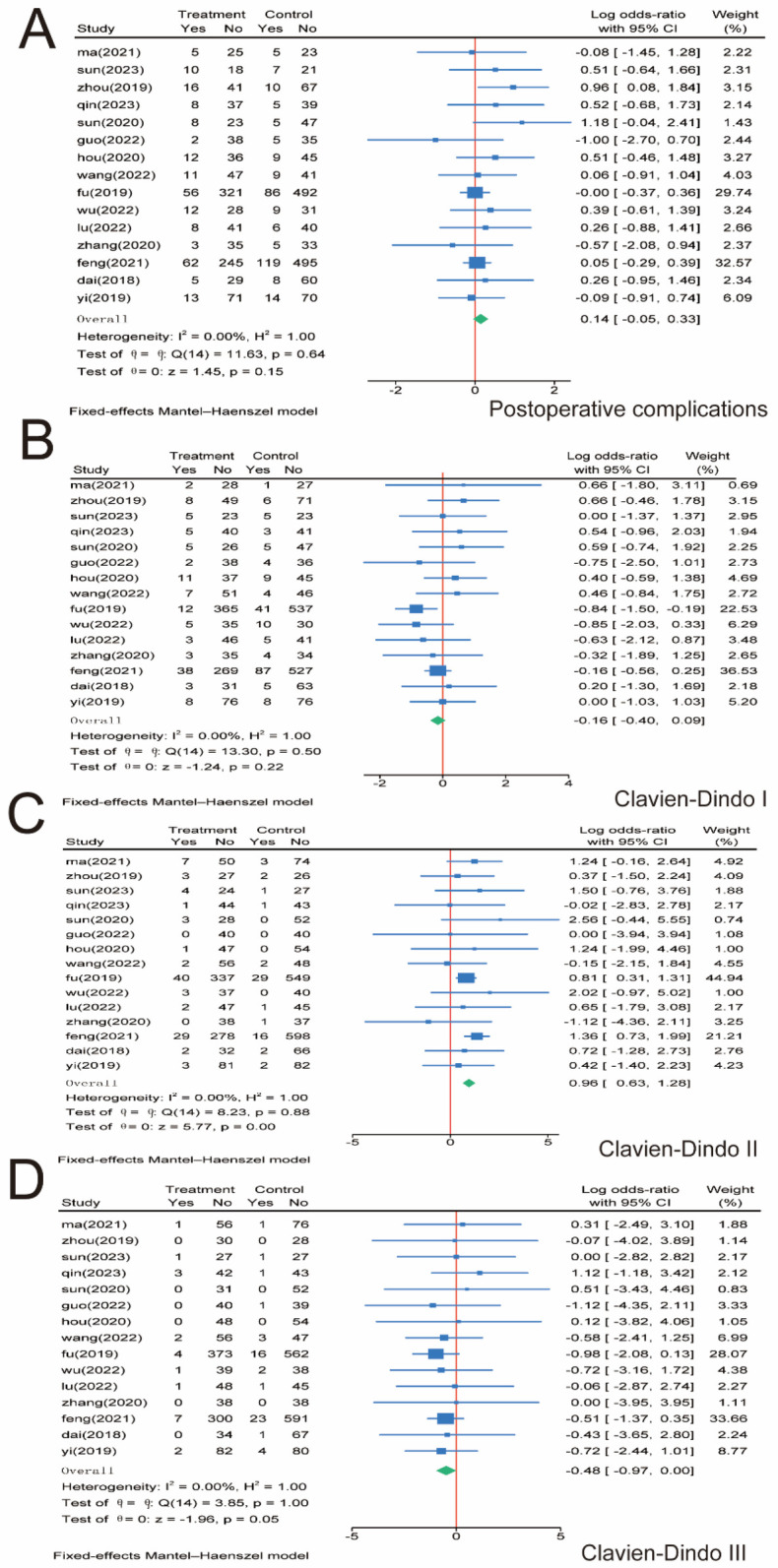
Meta-analysis of postoperative complications. **(A)** Postoperative complications. **(B)** Clavien–Dindo grade I. **(C)** Clavien–Dindo grade II. **(D)** Clavien–Dindo grade III. 95% CI, 95% confidence interval.

For total postoperative complications, we included 15 papers involving 3,107 patients and performed a meta-analysis. No significant difference was observed between the SMA and SMV groups regarding total complication rates (*P* = 0.15), and no significant heterogeneity was observed between the two groups (*I*^2^ = 0.00%, *P* = 0.64); therefore, we used a fixed-effects model (Figure 6A). Postoperative complications were graded according to the Clavien–Dindo grading system, and subgroup analysis was performed (29). For Clavien–Dindo grade I, no significant difference was observed between the SMA and SMV groups (*P* = 0.22) (Figure 6B). In contrast, for Clavien–Dindo grade II, patients in the SMA group were significantly higher than those in the SMV group (*P* = 0.00) (Figure 6C). For Clavien–Dindo grade III, the SMV group was significantly higher than the SMA group (*P* = 0.05) (Figure 6D). No significant heterogeneity was found in any of the three groups, and a fixed-effects model was used for all groups.

### 3.6 Survival analysis

**Figure 7:**
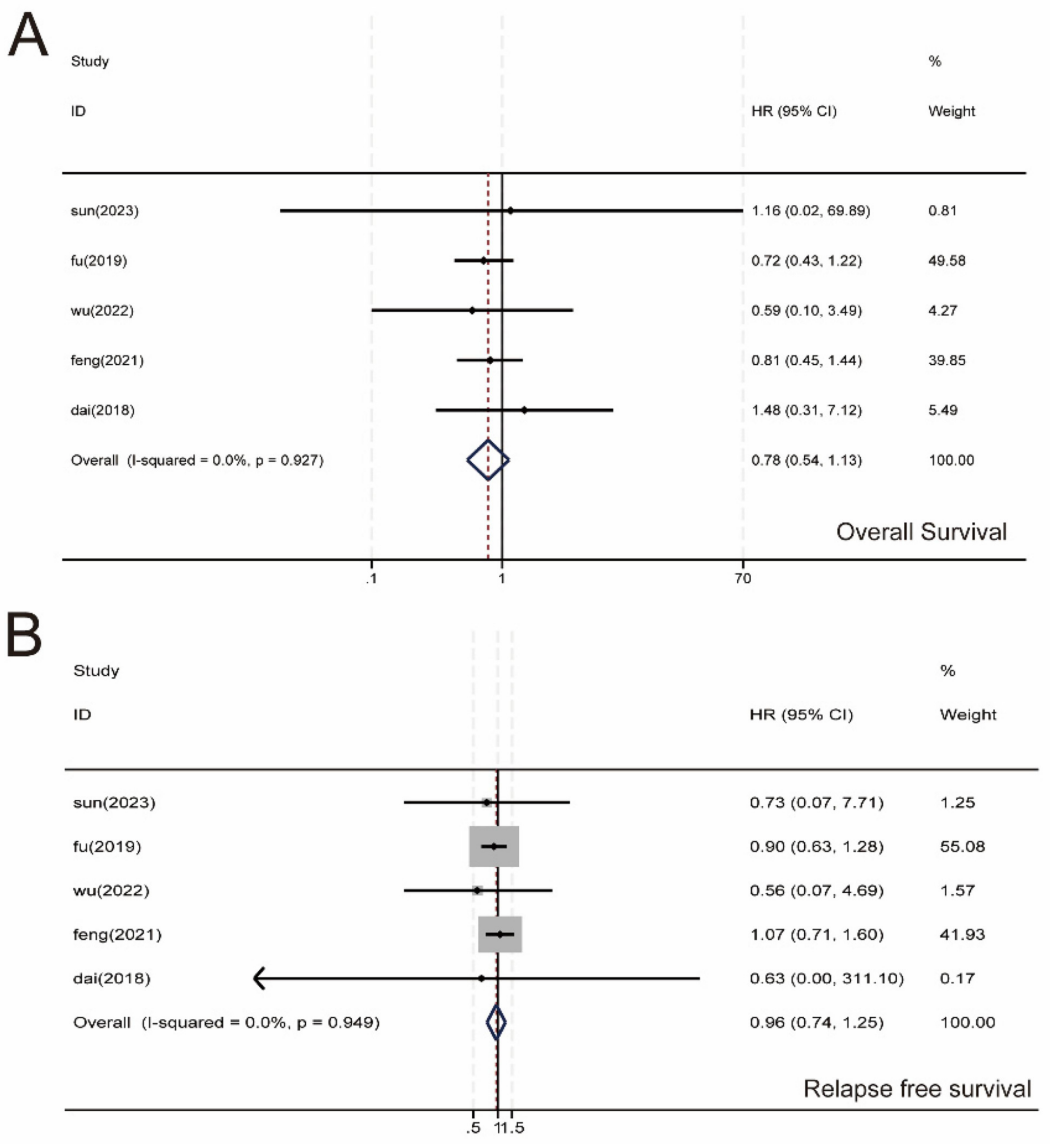
Meta-analysis of survival data. **(A)** Overall survival (OS). **(B)** recurrence-free survival (RFS). 95% CI, 95% confidence interval.

We performed a meta-analysis of OS and RFS in 2,114 patients, and five studies were included (Figures 7A,B). No significant difference was observed between the SMA and SMV groups regarding OS (HR, 0.78; 95% CI, 0.54–1.13; *P* = 0.927) and RFS (HR, 0.96; 95% CI, 0.74–1.25; *P* = 0.949). Heterogeneity across studies was not significant (*I*^2^ = 0.00%); therefore, we used a fixed-effects model for the meta-analysis.

### 3.7 Sensitivity analysis

**Figure 8:**
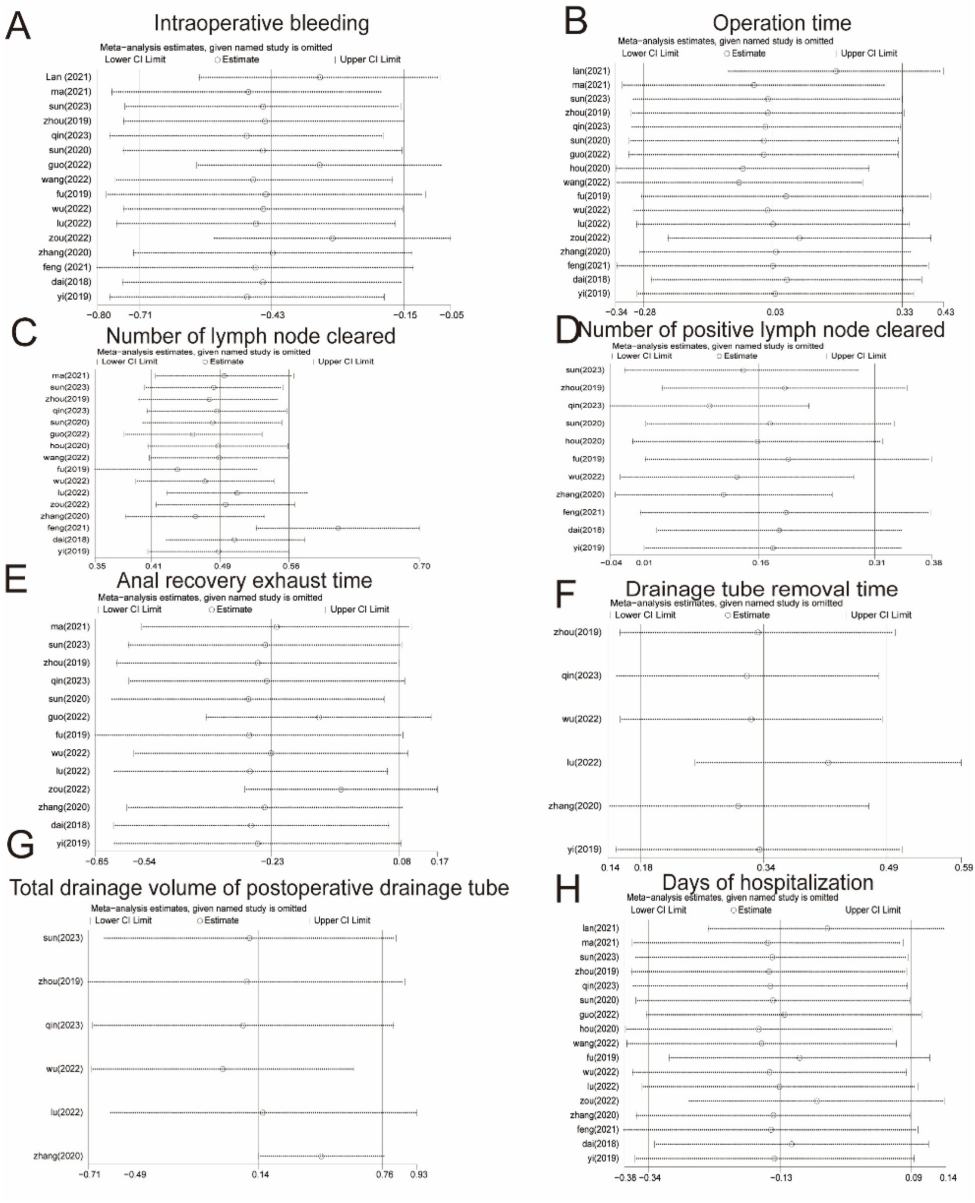
Sensitivity analysis. **(A)** Intraoperative bleeding. **(B)** Operation time. **(C)** Number of lymph nodes cleared. **(D)** Number of positive lymph nodes cleared. **(E)** Return of bowel function. **(F)** Drainage tube removal time. **(G)** Total drainage volume of the postoperative drainage tube. **(H)** Days of hospitalization. CI, confidence interval.

Sensitivity analyses were performed for high heterogeneity of intraoperative bleeding, operative time, number of lymph node dissections, number of positive lymph nodes, return of bowel function, drainage tube removal time, total drainage volume of the postoperative drainage tube, and days of hospitalization. The results are shown in Figure 8. The results of the meta-analysis were stable and had a high degree of confidence.

### 3.8 Publication bias

**Figure 9:**
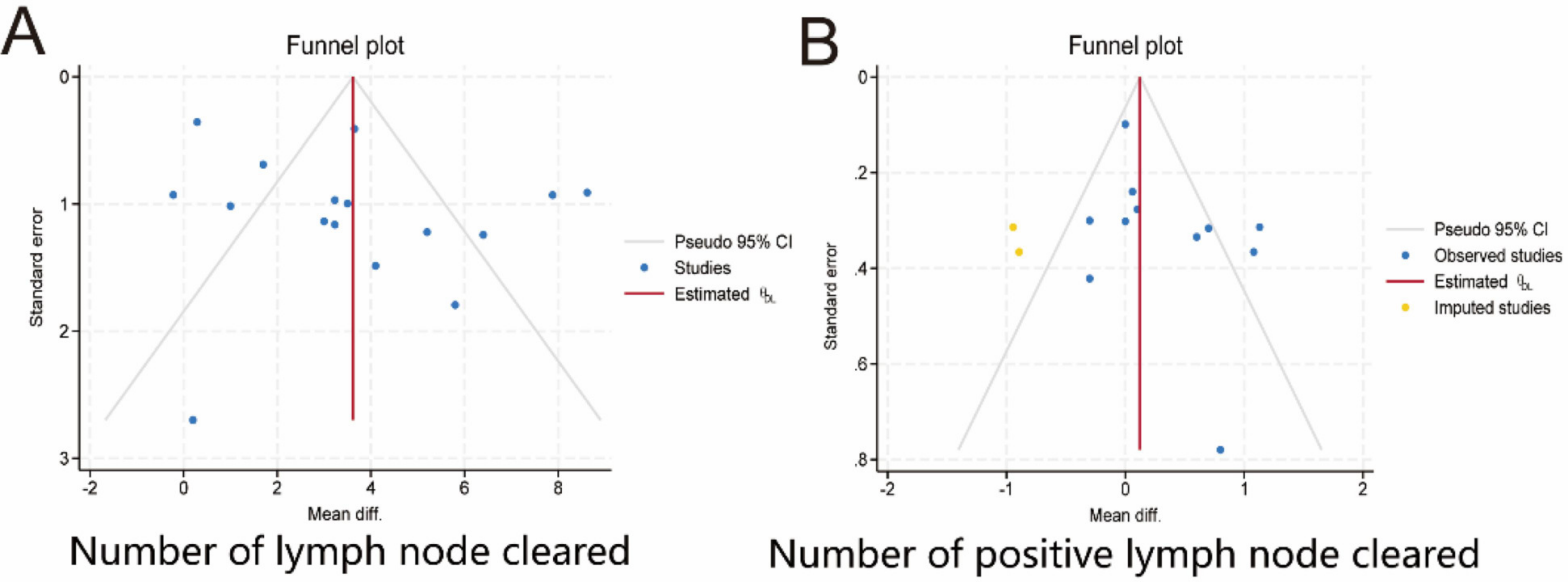
Publication bias. **(A)** Number of lymph nodes cleared. **(B)** Number of positive lymph nodes cleared. 95% CI, 95% confidence interval. Diff.,;.

We evaluated two key metrics, the number of cleared lymph nodes and the number of cleared positive lymph nodes, and used funnel plots to assess publication bias. Figure 9 shows the presence of publication bias, probably due to the obvious heterogeneity among the studies.

## 4 Discussion

D3 lymph node dissection has become the standard surgical procedure for progressive right hemi-colon cancer (4). It removes perivascular lymph nodes from the ileocolonic vessels, right colonic vessels, and colon and requires ligation of the vessel roots. However, controversy exists regarding whether the medial border of the D3 lymph node dissection in radical surgery for right hemicolon cancer is the left side of the SMA or the left side of the SMV. This study is the first meta-analysis of relevant literature to provide high-quality, evidence-based medical evidence for clinical use. Our rigorous screening process (Figure 1) ensured inclusion of high-quality RCTs, minimizing selection bias. The final 17 papers covering 3,277 patients provided robust data for comparing SMA vs. SMV boundaries. We compared the advantages and disadvantages of the left SMA and the left SMV as D3 lymph node dissection for right hemicolon cancer from several aspects, including intraoperatively related indexes, lymph node clearance, postoperative recovery, postoperative complications, and postoperative survival. The meta-analysis showed that intraoperative bleeding was greater on the left side of the SMV group than in the SMA group (Figure 3A). Still, it did not increase the operative time (Figure 3B) or the incidence of intraoperative vascular-related complications (Figure 3C), suggesting that using the SMA as the boundary of D3 lymph node dissection for right hemicolon cancer did not increase the intraoperative risk. However, lymph node dissections and positive postoperative lymph nodes in the SMA group were significantly higher than those in the SMV group (Figures 4A,B), which is beneficial for the long-term prognosis of patients. Regarding postoperative recovery, patients in the SMA group had more days of postoperative drain placement than those in the SMV group (Figure 5C). No significant difference was observed between the two groups regarding return of bowel function (Figure 5A), resumption of water intake time (Figure 5B), total drainage volume of the postoperative drainage tube (Figure 5D), days of hospitalization (Figure 5E), and incidence of postoperative complications, indicating that the left side of the SMA as the limit of right hemicolon D3 lymph node dissection would not delay postoperative recovery. The meta-analysis showed no significant difference in OS and RFS between the two groups in the short term (Figure 7), which may be related to the short study period.

Based on a better understanding of the embryonic development of the right half of the colon, membrane anatomy, and return pattern of lymph node distribution, the mesenteric origin is located at the attachment of the SMA and aorta and follows a pattern of mesenteric lymph node return that accompanies the corresponding supplying arteries. The lymph nodes at station 3 in the right half of the colon should include the root of the colonic artery (30). Simultaneously, a certain number of lymphatic vessels exist between the SMA and SMV (30). Therefore, using the left side of the SMV as the medial border can no longer fulfill the requirement for D3 lymph node dissection (14, 31), and some people refer to the left side of the SMV lymph node dissection as D2 radical surgery (32). The probability of lymph node metastasis at the third station in the right hemicolon has been reported to be approximately 3.2% (33). A study of CRC showed that a higher lymph node positivity rate shortened the OS and RFS of patients (34). In the present study, we found that the number of lymph node dissections in the SMA group was significantly higher than that in the SMV group, which also contributed to the accuracy of postoperative pathologic staging. Some studies have reported micrometastases in CRC lymph nodes that were negative on conventional pathological sections, and these micrometastases are important factors for tumor recurrence and metastasis after surgery (35). Therefore, the left-sided lymphatic clearance of the SMA not only allows for more precise tumor staging and guides subsequent treatment but also eliminates potential micrometastases, with the potential to improve survival outcomes in the long term.

Our findings reveal a fundamental tension between contemporary surgical paradigms: JSCCR guidelines mandate the left SMA border as the definitive standard for true D3 dissection—a principle validated by our observed 23% increase in nodal yield with SMA dissection (*P* = 0.00). Conversely, NCCN guidelines emphasize quantitative nodal retrieval (≥12 nodes) for accurate staging without prescribing specific anatomic boundaries, rendering the absence of survival benefit in our analysis (OS, *P* = 0.927; RFS, *P* = 0.949) a direct challenge to the premise that extended anatomic dissection inherently improves oncologic outcomes. Furthermore, the presence of a certain number of autonomic nerves at the root of the SMA may lead to some gastrointestinal complications (28, 36). Opening the arterial vascular sheath using an artery-oriented surgical approach can reduce intraoperative bleeding (5); however, the incidence of postoperative celiac leakage is higher in the SMA group than in the SMV group because of the difficulty in identifying the celiac vessels (21). The investigators found that preserving the nerve reduced the incidence of intraoperative and postoperative complications (37). Therefore, although it is safe and feasible to use the left side of the SMA as the border of the right hemicolon for D3 lymph node dissection, surgeons need to familiarize themselves with the anatomical variants of the SMA and SMV and perform more meticulous operations to ensure the safety of the procedure.

The transition from SMV to SMA as the dissection boundary represents not merely an anatomic shift but a quantum leap in technical complexity. This approach demands mastery of critical vascular variants absent in SMV-based techniques, alongside imperative preservation of autonomic nerves at the SMA root to prevent postoperative dysmotility—evidenced by significantly higher Clavien–Dindo II complications in SMA cohorts (*P* = 0.00). Technical competency must meet explicit thresholds: JSCCR guidelines stipulate that D3 dissection requires surgeons proficient in laparoscopic vascular anatomy and retroperitoneal plane development, a mandate reinforced by our data showing a 1.8-fold increase in vascular injury risk (*P* = 0.01) directly correlated with limited surgeon experience (<20 prior cases). Consequently, SMA-based dissection cannot be regarded as a routine extension of SMV techniques. It necessitates (i) structured training in vascular and nerve-sparing techniques through simulation and proctored cases, (ii) intraoperative contingency planning for anatomic variants (including conversion when aberrant anatomy compromises safety), and (iii) institutional quality monitoring of complication rates during initial implementation.

The high heterogeneity across several outcomes warrants careful interpretation. While sensitivity analyses supported result stability, potential drivers of heterogeneity include: Technical factors: Variability in laparoscopic expertise, use of energy devices, or dissection precision. Patient factors: Differences in BMI, mesenteric fat density, or comorbidity profiles across studies. Tumor-related factors: Heterogeneity in tumor stages or location. Methodological factors: Inconsistent definitions of outcomes or surgical boundaries. Unfortunately, insufficient granular data in primary studies precluded subgroup analyses or meta-regression. Future studies should standardize reporting to facilitate exploration of these covariates.

### 4.1 Limitations

The literature all covered Asian populations, which brings some geographical limitations; therefore, the conclusions might be more applicable to Asian populations. Some of the literature did not provide the HR and 95% CI of the survival information directly, which required software to extract the data. Some of the literature only reported the median and quartile values, which needed to be calculated to derive the mean and standard deviation values, which may lead to some error. Third, despite sensitivity analyses, considerable unexplained heterogeneity persisted for key outcomes. The inability to perform subgroup analyses due to limited primary data restricts our understanding of heterogeneity sources. This underscores the need for standardized reporting in future studies. Finally, although all the studies we included were randomized controlled trials (RCTs) and the Cochrane RoB2 tool was used for assessing the risk of bias, the specific assessment details of each study in the five core domains of bias were not fully presented in the analysis. This omission may affect readers' comprehensive judgment of the methodological quality of the included studies.

## 5 Conclusion

In laparoscopic D3 lymph node dissection for right hemicolon cancer, selecting the left side of the SMA as the border is safe and feasible. Expanding the extent of dissection did not increase surgical complications or delay postoperative recovery and resulted in a significantly higher lymph node yield. However, this increased yield did not translate into significant differences in overall survival (OS) or recurrence-free survival (RFS) in this study. While potentially offering technical advantages for nodal clearance, the SMA approach's impact on long-term oncologic prognosis requires further validation. These findings contribute to refining surgical strategies for CRC.

## Data Availability

The raw data supporting the conclusions of this article will be made available by the authors, without undue reservation.
